# Fixation Characteristics in Highly Myopic Eyes: the Shanghai High Myopia Study

**DOI:** 10.1038/s41598-019-42895-3

**Published:** 2019-04-24

**Authors:** Xiangjia Zhu, Wenwen He, Keke Zhang, Yinglei Zhang, Qi Fan, Yi Lu

**Affiliations:** 1 Department of Ophthalmology, Eye and Ear, Nose, and Throat Hospital, Fudan University, Shanghai, China; 2 Eye Institute, Eye and Ear, Nose, and Throat Hospital of Fudan University, 83 Fenyang Road, Shanghai, 200031 People’s Republic of China; 3 NHC Key Laboratory of Myopia (Fudan University), Shanghai, 200031 People’s Republic of China; 4 Key Laboratory of Myopia, Chinese Academy of Medical Sciences, Shanghai, People’s Republic of China; 5 Shanghai Key Laboratory of Visual Impairment and Restoration, Shanghai, 200031 People’s Republic of China

**Keywords:** Refractive errors, Epidemiology

## Abstract

We enrolled 500 highly myopic eyes and 50 controls in this hospital-based prospective cohort study. The fixation ellipse angle and area in terms of the bivariate contour ellipse area (BCEA) were measured with Macular Integrity Assessment microperimetry. Optic disc tilt and rotation were evaluated with retinal images. The associations between fixation and optic disc changes were assessed. Both 63% and 95% BCEA correlated positively with axial length (AL) (both r = 0.230, P = 0.001) in highly myopic group, and were significantly higher than the control group (both P < 0.001). The direction of fixation ellipse presented clockwise rotation in the right eyes and anti-clockwise rotation in the left eyes with the increase of AL in highly myopic group (AL ≥30 vs <30 mm: OD 76.12 ± 51.17°: vs 90.60° ± 51.28°, P = 0.029; OS 94.73 ± 57.45° vs 87.82 ± 55.15°, P = 0.371). The angle between the long axis of the fixation ellipse and the long axis of the optic disc (Angle_F−D_) distributed in various directions: 0–30° (34.6% almost parallel) ≈60–90° (34.4% almost vertical) >30–60° (31% oblique). Angle_F−D_ increased slightly with the AL (r = 0.105, P = 0.024). In highly myopic eyes, fixation stability decreased with the AL, and superior rotation of the fixation ellipse increased with AL. The long axis of fixation ellipse and the long axis of optic disc became less parallel to each other with increasing AL. Our data may provide clues for improvement of fixation evaluation designs of biometric instruments.

## Introduction

High myopia is a major cause of visual impairment throughout the world and it is especially prevalent in East Asia^1,2^. The characteristic ocular changes in high myopia include an excessive increase in the axial length (AL), the deformation of the posterior segment in the form of staphyloma, and the development of a range of retinal and choroidal lesions^3,4^. Therefore, the fundus conditions in highly myopic eyes are usually poor.

A poor fundus can result in poor visual function, and the ability to maintain steady fixation may be impaired in high myopes. Previous studies have shown that the eye is not truly static when staring at a point target^5,6^. There are always fixational eye movements, such as microsaccades, drift, and tremor, which keep the retina in motion^5,6^. Healthy eyes use central fixation to maintain the target, and the magnitudes of the eye movements are small. However, these eye movements are larger in highly myopic eyes, as indicated by the fixation ellipse area, according to our previous study^7,8^.

We also noted that the fixation ellipse has an orientation that indicates the distribution of the majority of fixation points. As accurate measurements of axial length in highly myopic eyes have always been difficult^9^, significant refractive errors are often seen in these patients after cataract surgery^10,11^. Our previous data has indicated the significance of steady fixation in AL acquisition^7,8^. The new generation of IOLMaster 700 has also included the fixation evaluation function to assess whether the patient fixates with the fovea during examination. However, it only tells the clinician one macular location at the time of data acquisition, which might not reflect those most frequently used macular locations if the patients’ fixation are unsteady. Thus, the direction of fixation ellipse calculated by MAIA microperimetry may provide us with extra information on the trace of those most frequently used macular loci during fixation, which will be useful in those with poor fundus, such as highly myopic cases. According to this direction of fixation ellipse, we may be able to correct data measured by the IOLMaster in the future, or to obtain the “most frequently used macular loci corrected AL” in eyes with high myopia. However, up to now, very few studies have investigated this direction of fixation ellipse in myopic eyes.

Because staphyloma forms at the posterior pole in highly myopic eyes, these eyes also tend to develop various characteristic features of the optic disc, including optic disc tilt, rotation, and parapapillary atrophy (PPA)^12–14^. Previous studies of myopic disc changes have mainly focused on glaucomatous eyes, in which disc torsion and PPA are often used as predictors of visual field defects. However, according to the Blue Mountains Eye Study^15^, glaucoma was only present in 4.2% of eyes with low myopia and in 4.4% of eyes with moderate-to-high myopia. Therefore, for the majority of high myopes, many factors other than glaucoma shall also be considered. Previous studies have also managed to judge the direction of corneal astigmatism from the features of the optic disc^16^, as the latter is easier to visualize with a funduscope, and we speculate if there are some correlations between the fixation ellipse direction and the optic disc direction, as the two seems anatomically and functionally related.

The Macular Integrity Assessment™ (MAIA) microperimetry system (Centervue, Padova, Italy) combines functional and structural measurements of the macula. With an advanced eye tracking technique, the MAIA system permits fixation eye movements to be monitored precisely in real time during an examination^17^. The aims of this study were to characterize the fixation in high myopes with the MAIA microperimetry system and to investigate its anatomical association with optic disc changes.

## Results

The characteristics of the included subjects are shown in Table 1. The average age was 61.34 ± 8.82 years, and 201 subjects were male and 299 were female. The mean AL in the highly myopic group was 29.54 ± 2.26 mm, with a range of 26.00–36.42 mm. The mean AL in the control group was 24.95 ± 1.08 mm, with a range of 22.78–25.89 mm. Notably, in the highly myopic group, 53.6% (268/500) of the eyes had tilted discs, and in the control group, only 10% (5/50) of the eyes had tilted discs (Chi-square test, P < 0.001). In the highly myopic group, the disc was significantly rotated in 45.4% (227/500) of eyes and 67.8% (339/500) presented with inferior rotation In the control group, the dict was significantly rotated in only 16.0% (8/50) of the eyes.

**Table 1:** Demographic Characteristics of participants.

Parameter	Highly Myopic Group	Control Group	P value
Age (year)	61.34 ± 8.82	62.20 ± 7.44	0.505
Gender (Male/Female)	201/299	21/29	0.805
Eye (Right/Left)	262/238	22/28	0.257
Pre-UCVA (logMAR)	1.22 ± 0.68	0.84 ± 0.34	<0.001
Pre-BCVA (logMAR)	0.89 ± 0.64	0.80 ± 0.33	0.355
AL (mm)	29.54 ± 2.26	24.95 ± 1.08	<0.001
IOP (mmHg)	15.55 ± 3.39	15.09 ± 2.86	0.359
Optic Disc Tilt			
Tilted disc/disc without tilt, no (%)	268 (53.6)/232 (46.4)	5 (0.1)/45 (0.9)	<0.001
Tilt ratio	1.34 ± 0.23	1.15 ± 0.11	<0.001
Optic Disc Rotation			
Superior/inferior	161 (32.2)/339 (67.8)	16 (32.0)/34 (68.0)	0.977
Significant/insignificant	227 (45.4)/273 (54.6)	8 (16.0)/42 (84.0)	<0.001
Rotation degree (°)	8.21 ± 31.32	2.48 ± 15.60	0.031

Pre-UCVA = preoperative uncorrected visual acuity; Pre-BCVA = preoperative best corrected visual acuity; AL = axial length; IOP = intraocular pressure.

According to the OCT data, in highly myopic groups, 47 eyes (9.4%) had retinoschisis but without macular hole and 14 eyes (2.8%) had mild retinal edema. No eye had choroidal neovascularization and macular atrophy. In the control group, no obvious abnormality was observed in OCT images. 63% BCEA and 95% BCEA were both higher in the highly myopic group than the control group (Student’s t-test, 63% BCEA: 5.53 ± 7.04 vs 1.15 ± 0.51, P < 0.001; 95% BCEA: 3.41 ± 1.51 vs 16.56 ± 21.10, P < 0.001). The mean postoperative BCVA of the highly myopic group was 0.19 ± 0.23 logMAR. All patients got BCVA no less than 20/200, and 83.4% patients got BCVA better than 20/40. The 95% BCEA positively correlated with postoperative BCVA in the highly myopic group (Pearson’s analysis, r = 0.387, P < 001).

**Figure 1:**
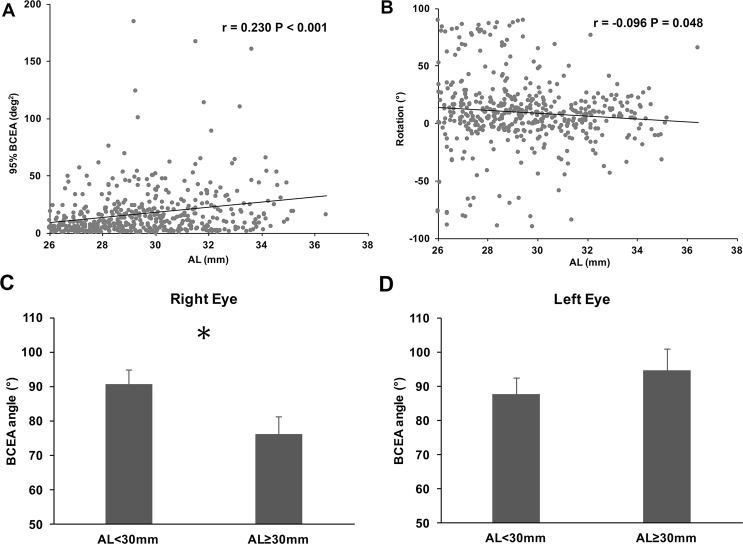
Characteristics of fixation and changes in disc rotation with axial length. (**A**) The 95% BCEA correlated positively with axial length (Pearson’s analysis, 95% BCEA: r = 0.230, P < 0.001). (**B**) Optic disc rotation correlated weakly negatively with axial length (Pearson’s analysis, r = −0.096, P = 0.048). (**C**) Comparisons of fixation ellipse angle between different AL groups in right eyes (Student’s t-test, *P = 0.029). (**D**) Comparisons of fixation ellipse angle between different AL groups in left eyes. (Student’s t-test, P = 0.371). BCEA, bivariate contour ellipse area.

**Figure 2:**
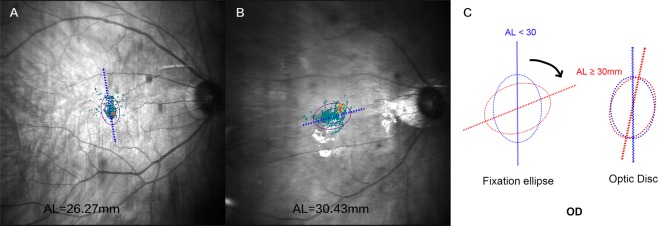
Representative images of the direction of fixation ellipse with different ALs. (**A**) A 58-year old man with AL of 26.22 mm and fixation ellipse angle of 97.4° in the right eye. (**B**) A 50-year old man with AL of 30.43 mm and fixation ellipse angle of 13° in the right eye. (**C**) The direction of fixation ellipse presents superior rotation with the increase of AL and it rotates more than the optic disc. AL, axial length.

The patients in the highly myopic group were then divided into three groups according to their AL. Table 2 shows the fixation and optic disc changes in each group. Statistically significant differences were detected among the three groups in terms of 63% BCEA and 95% BCEA (ANOVA, P < 0.001 and P < 0.001, with post hoc Tukey test; 63% BCEA: Group 1 vs 2, P < 0.001, Group 1 vs 3, P < 0.001, Group 2 vs 3, P = 0.363; 95% BCEA: Group 1 vs 2, P < 0.001, Group 1 vs 3, P < 0.001, Group 2 vs 3, P = 0.362). Figure 1 shows that in the highly myopic group, 95% BCEA positively correlated with AL (Pearson’s analysis, 95% BCEA: r = 0.230, P < 0.001, similar data were found for 63% BCEA: r = 0.230, P < 0.001), and optic discs became less inferior rotated with increase of AL (Pearson’s analysis, r = −0.096, P = 0.048). However, no such significant relationship was found in the control group (Pearson’s analysis, all P > 0.05) The variation in the optic disc rotation also became much smaller as the AL increased in the highly myopic group, as indicated by the standard deviation (Table 2) and the scatter plot (Fig. 2). On average, the direction of fixation ellipse presented superior rotation with the increase of AL in the highly myopic group (AL ≥ 30 vs < 30 mm: OD 76.12 ± 51.17° vs 90.60° ± 51.28°, P = 0.029; OS 94.73 ± 57.45°vs 87.82 ± 55.15°, P = 0.371, Figs 1 and 2).

**Table 2:** Fixation and optic disc changes in different axial length groups.

Parameter	26 ≤ AL <28 mm	28 ≤ AL <30 mm	AL ≥ 30 mm	P value
Number of eyes	165	157	178	0.364
63% BCEA (deg^2^)	3.14 ± 3.70 (0.2–20.5)	6.16 ± 7.72 (0.3–61.7)	7.2 ± 8.22 (0.2–55.8)	<0.001
95% BCEA (deg^2^)	9.41 ± 11.09 (0.6–61.5)	18.44 ± 23.12 (0.9–184.8)	21.57 ± 24.61 (0.3–167.1)	<0.001
Rotation (°)	9.56 ± 38.59 (−88.5–89.5)	12.25 ± 30.85 (−90–89.9)	5.29 ± 20.74 (−83.8–76.9)	0.071
Tilt ratio	1.3 ± 0.21 (1.01–2.00)	1.36 ± 0.2 (1.00–1.96)	1.35 ± 0.25) (1.00–2.32)	0.272

BCEA = bivariate contour ellipse area; deg^2^ = degrees-squared.

**Figure 3:**
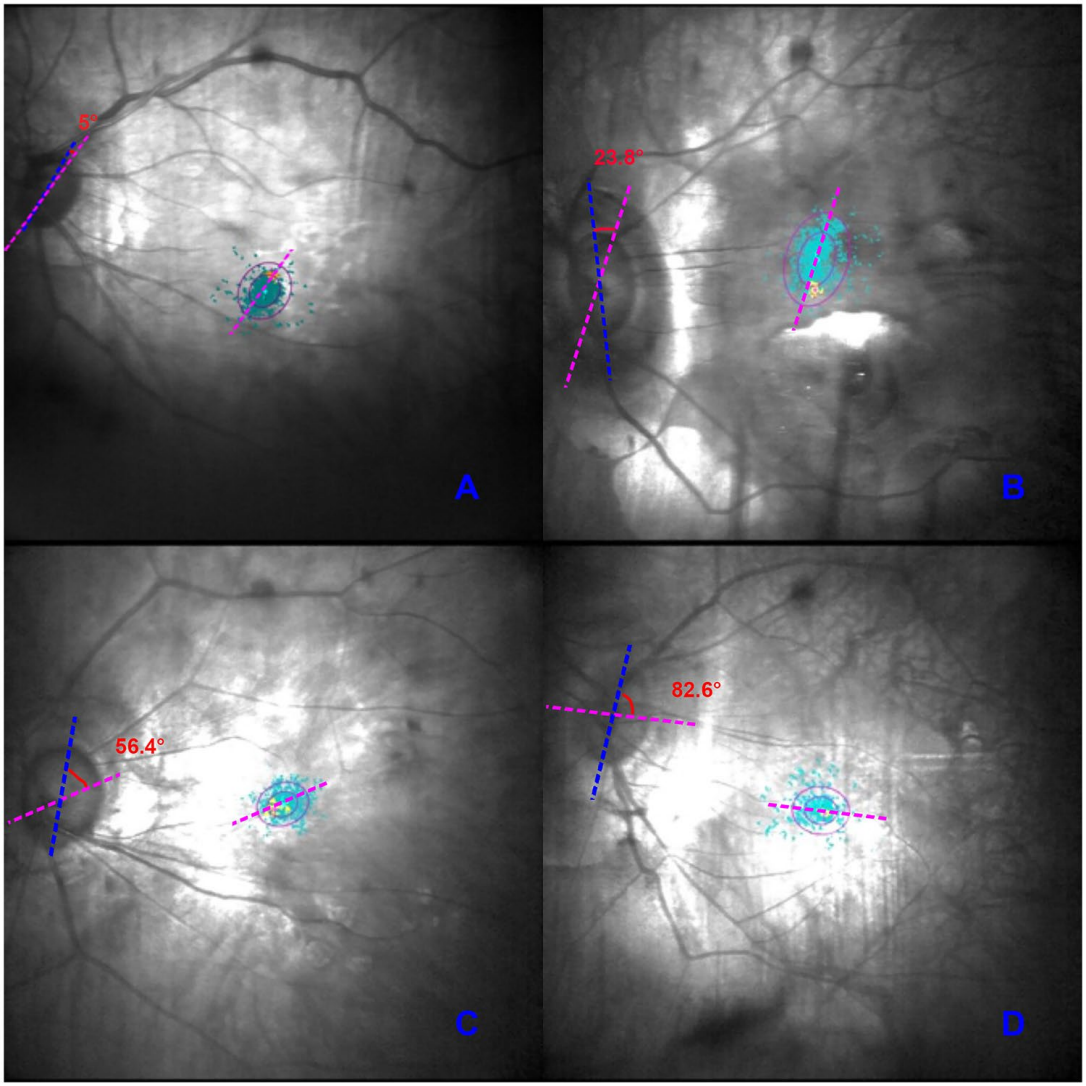
Representative images of different Angle_F−D_. Angle_F−D_ is defined as the acute angle between the long axis of the fixation ellipse and the long axis of the optic disc. The purple-red line represents the long axis of the fixation ellipse and the blue line represents the long axis of the optic disc. The angle shown in red is Angle_F−D_. Panels from A to D show representative images of Angle_F−D_ from small to larger. BCEA, bivariate contour ellipse area.

Figure 3 shows representative Angle_F−D_ values, the acute angles between the long axis of the fixation ellipse and the long axis of the optic disc. The average Angle_F−D_ in the highly myopic group was 44.66 ± 26.39°, and it ranged from 0° to 90.00°. The average Angle_F−D_ in the control group was 43.88 ± 29.90°, and it ranged from 0.1° to 88.40°. There was no siginificant difference between the two groups (Student’s t-test, P = 0.844). Interestingly, in the highly myopic group, the BCEA and optic disc were not actually parallel in most cases, contrary to our expectation. This angle can be variable. The distribution of Angle_F−D_ in different directions: 0–30° (34.6% almost parallel) ≈60–90° (34.4% almost vertical) >30–60° (31% oblique). On average, Angle_F−D_ increased slightly with AL (Pearson’s analysis, r = 0.105 P = 0.024).

## Discussion

The eye is not totally static when staring at a target. Microsaccades, drift, and tremor are always present, which keep the retina in motion^5,6^. When the macular (especially the foveal) conditions are poor, patients tend to have poor fixation stability or adopt alternate preferred retinal loci of fixation, eccentric to the fovea. Mallery *et al*.^18^ used the confocal scanning laser ophthalmoscope (cSLO)-based retinal tracking capability of Heidelberg OCT to assess fixation abnormalities, and investigated the fixation pattern relative to the ganglion cell complex in patients with optic neuropathy (ON). They found that fixation preferences, quantified as fixation instability, were altered in patients with ON. Eyes with ON had almost 2-3-fold more fixation instability than normal control eyes. Therefore, fixation stability correlates with visual function and affects the performance of visually demanding tasks.

Fixation abnormalities in patients with high myopia are becoming increasingly recognized as well. According to our previous study, highly myopic eyes generally show unstable fixation stability^7,8^. These eyes may have some degree of central fixation, but the frequency of their fixation targeting on the fovea is much lower than in emmetropic controls, as indicated by the BCEA, which is the area of the ellipse containing most of the fixation positions registered during the evaluation process. The instability of fixation also increases as the AL increases, indicating a reduction in central fixation.

Although cases with severe fundus conditions were excluded from the present study, fixation reduction attributable to the thinning of the retina and the retinal pigment epithelium, resulting from the extreme extension of the posterior pole of the eyeball^19^, was unavoidable. However, the r of the correlation between fixation and the AL was minor, indicating that there are other factors affecting fixation stability except AL, which need to be further studied.

Another interesting finding of this study is that the direction of fixation ellipse rotates superiorly as the AL increases, which might be a compensatory response to the general inferior rotation of the eye with long AL to maintain the visual axis within the pupil boundary. The angle of the fixation ellipse indicates the most likely track of eye movements during examination, which may provide important clues for the future correction of AL measured by instruments like IOLMaster in highly myopic cases. IOL power calculation according to the AL corrected with the main track of eye movements may be more acceptable by the patients after surgery as macular loci on this track (along fixation ellipse angle) are more often used.

Tilt and rotation are two common characteristic changes in the optic discs of highly myopic eyes. Recently, in addition to the tilt inferred from the ratio between the longest and shortest diameters of the optic disc, OCT has been used for the direct measurement of the cross-sectional tilt of the optic disc. But a comparison of the two measures indicated that the outcomes are not interchangeable, because their correlation is only low to moderate^20^. Therefore, we chose the more common and easier way of assessing tilt, with a photograph-based method.

One advantage of the present study was the sampling of relatively old subjects with high myopia (average age, 61.34 years). As we know, high-myopia-related structural changes in the posterior pole can be age- and time-dependent, so the eyeballs in our study may have been fully “developed” than those in previous studies, which mainly examined teenagers^21^ or young people^13,22^. In total, 53.6% of the subjects in our study had a tilted disc and 45.4% had significant rotation. These rates are significantly higher than those in studies that targeted younger subjects^14,23^. In our study, the tilt ratio was 1.34 ± 0.23, which is significantly higher than that reported in the study of Sung *et al*.^14^. The average rotation was 8.21° and 67.8% of eyes presented with inferior rotation. These parameters were also significantly higher than the values of 3.44° and 33.18%, respectively, in Sung *et al*.’s study^14^, which targeted a sample with an average age of 27.94 years and lower-level myopia. Even more interestingly, the variation in optic disc rotation became smaller as the eyeball became longer, which means that in eyes with extremely long axes, the magnitude of rotation decreases.

Previous studies of the association between the direction of the fixation ellipse and the optic disc are quite rare. It may be assumed that anatomically, the long axes of the fixation ellipse and the optic disc shall be parallel to maintain the uniform function of the eye. However, this was not the case according to the present study. Only one third of the highly myopic eyes had an Angle_F−D_ of 0–30° (parallel), whereas in one third of eyes, it was 60–90° (vertical), and in the other one third, it was intermediate between the two. The exact mechanism underlying the variation in Angle_F−D_ remains unclear. We speculate that it may be attributable to the nonuniform configuration or thickness of the macula, induced by the overextension of the eyeball and the variations in the location and depth of the posterior staphyloma. Macular loci that function better may be selected for fixation. On average, the acute angle between the long axis of the fixation ellipse and the long axis of the optic disc increases with AL, indicating that the two became even less parallel in extreme myopia.

Because our study targeted an older highly myopic population, it is very difficult to eliminate the influence of cataract because myopia itself is a recognized risk factor for early-onset of cataract^24^. Thus, fixation was evaluated after surgery to avoid the impact of a cloudy lens on the imaging quality. Our study adds detailed information to the current knowledge of fixation in older highly myopic eyes: the decrease of fixation stability with AL warns cataract surgeon to be careful when carrying out preoperative IOLMaster examination on highly myopic eyes with extremely long AL, and the various distribution of Angle_F−D_ reminds us that we should not simply judge the direction of fixation ellipse on the orientation of optic disc without carrying out microperimetry, especially in extremely highly myopic eyes.

To conclude, we investigated the characteristics of fixation in an older highly myopic sample and found that with increase of axial length, fixation instability increases, and direction of fixation ellipse rotates superiorly in both eyes. The fixation ellipse and optic disc were not parallel in most cases, contrary to our expectation, and the acute angles between the two increased slightly with axial length. Our data may provide clues for improvement of fixation evaluation designs of biometric instruments in the future.

## Materials and Methods

### Patient and public involvement

The Shanghai High Myopia Study is a hospital-based prospective cohort study. The study includes highly myopic patients and control patients who were scheduled for cataract surgery at the Eye and Ear, Nose, and Throat (EENT) Hospital of Fudan University since October 2015. All the subjects underwent detailed preoperative examinations and careful postoperative follow-ups.

For this study, 500 individuals with the AL of ≥26 mm in both eyes and an age of 30+ years were included as the highly myopic group. For the control group, 50 individuals with the AL of <26 mm in both eyes and an age of 30+ years were included. Those with severe myopic fundus pathology, such as choroidal neovascularization, severe macular atrophy (Category 4 of myopic maculopathy according to the classification of Ohon-Matsui *et al*.^4^ and with best corrected visual acuity [BCVA] less than 20/200), or macular hole were excluded. One eye of each participant was randomly selected for investigation. The Institutional Review Board of the EENT Hospital of Fudan University approved the protocol of this study. All procedures adhered to the tenets of the Declaration of Helsinki. The participants were informed of the study objectives, and signed informed consent was obtained from all of them. The study was registered at www.clinicaltrials.gov (accession number NCT03062085).

### Preoperative and postoperative examinations

The preoperative examinations included the routine assessment of visual acuity, fundoscopy, tonometry, corneal topography (Pentacam HR, OCULUS Optikgeräte GmbH, Wetzlar, Germany), B scans, optical coherence tomography (OCT) macular scan (Zeiss Cirrus HD-OCT 5000, Carl Zeiss AG, Jena, Germany), and the measurement of AL (IOLMaster 500, version 7.7, Carl Zeiss AG). All patients underwent successful cataract surgery with the same type of intraocular lens implanted. Follow-up examinations were also performed one month after cataract surgery, including the assessment of visual acuity, manifest refraction, tonometry, OCT macular scan, fundus photography, and MAIA microperimetry.

### Fixation evaluation

**Figure 4:**
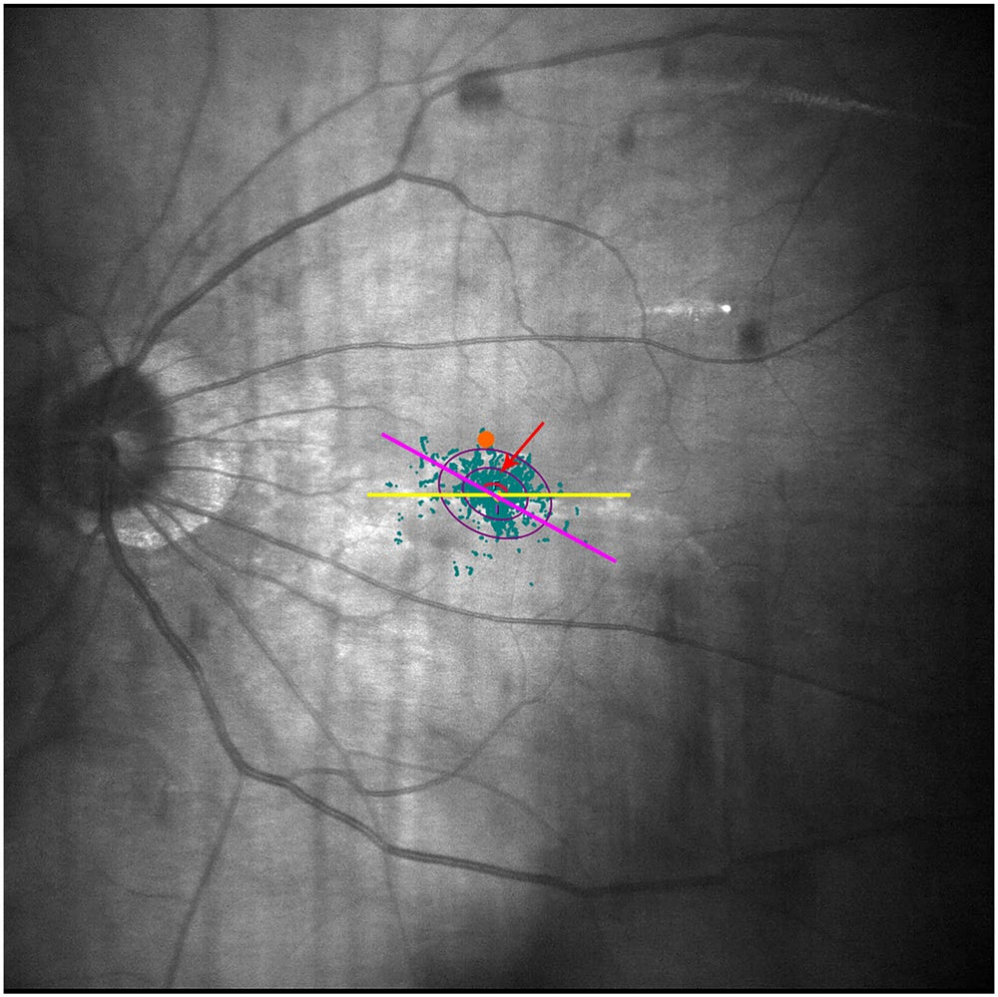
Illustration of the examination of fixation stability with the Macular Integrity Assessment™ (MAIA) microperimeter system. The inner and outer purple ellipses refer to the 63% BCEA and 95% BCEA, respectively, which represent the areas (in degrees-squared, deg^2^) of the ellipses containing 63% and 95% of the fixation positions registered during the measurement procedure. The purple-red line represents the long axis of the fixation ellipse and the red arrow indicates the fixation ellipse angle. The long axis of the fixation ellipse indicates the most likely track of eye movements during examination. The orange spot was an example of a fixation point far away from this track, which may induce significant refractive error after cataract surgery and is needed to be corrected. BCEA, bivariate contour ellipse area.

Fixation was investigated with the MAIA microperimetry system without glasses. The system could automatically adjust refractive error ranging from +10 D to −15 D during measurement. In the MAIA system, fixation is controlled by eye trackers that detect fixation losses as misalignments between the direction of the central fixation stimulus and the gaze. During the examination, the patient is asked to stare at the fixation stimulus, which consists of a red circle with a diameter of 1°. The eye trackers then record the points of fixation during the examination. The device automatically generates two parameters of fixation: the bivariate contour ellipse area (BCEA), which represents the area in degrees squared (deg^2^) of the ellipse containing most of the fixation positions registered during the measurement procedure; and the fixation ellipse angle, the angle between the long axis of the ellipse and the horizontal reference line (Fig. 4). BCEA is normally calculated by considering 63% or 95% of the fixation points used in the system.

### Measurement of optic disc tilt and rotation

Ultrawide-field retinal images focusing on the optic disc and macula were obtained with a nonmydriatic ultrawide-field scanning laser ophthalmoscope (Optomap 200Tx; Optos Plc., Dunfermline, Scotland, UK) with standard settings. The measurements of the optic disc tilt and rotation were made as previously described^14,25^. Optic disc tilt was measured as the ratio between the longest and shortest diameters of the optic disc. The optic disc was deemed to be tilted in those eyes with tilt ratios >1.30. Rotation was defined as the deviation of the long axis of the optic disc from the reference line, which was positioned at 90° to a horizontal line connecting the fovea and the center of the optic disc. The angle between the long axis of the optic disc and the reference line was deemed the degree of rotation. Superior rotation is given as a negative value, and inferior rotation as a positive value. The optic disc was classified as having significant rotation when the degree of rotation exceeded 15°.

The angle between the long axis of the fixation ellipse and the long axis of the optic disc (Angle_F−D_) was measured as follows. For each selected eye, the MAIA image with the fixation ellipse was exported to a desktop computer as a TIFF image file. The file was then processed with the Adobe Photoshop 2015 CC software (Adobe Systems, San Jose, CA). The long axis of the fixation ellipse and the long axis of the optic disc were marked on each image. An additional line that crossed the center of the optic disc, parallel to the long axis of the fixation ellipse, was then added. The acute angle between the long axis of the optic disc and the added line was calculated by the software.

### Statistics

Agreement on the long axis of optic disc, optic disc tilt ratio and the degree of rotation between two observers was assessed with the Bland–Altman method, which plots their means against their differences (Supplementary Fig. 1). The limits of agreement were calculated as the mean difference of two measurements ±1.96 standard deviations of the difference, according to previous studies^14,26^.

All analyses were performed with SPSS version 11.0 (SPSS Inc., Chicago, IL, USA) and all data are presented as means ± standard deviations. Pearson’s correlation test was used to determine the relationships between continuous variables. Student’s t-test was used to compare the differences in continuous variables. Chi-square test was used to compare the difference in categorical variables. One-way analysis of variance (ANOVA) was used to compare the differences in continuous variables among groups and Tukey’s test was used for post hoc analyses. P values < 0.05 were considered statistically significant.

## Supplementary information

Supplementary Figure 1

## Data Availability

All data will be available if requested.
