# Supplementary material for: Fixation Characteristics in Highly Myopic Eyes: the Shanghai High Myopia Study
Source: Sci Rep. 2019 Apr 24;9:6502. doi: 10.1038/s41598-019-42895-3 (PMC6482140; doi:10.1038/s41598-019-42895-3)
Supplement: Supplementary file 1 — Supplementary Figure 1 [file 41598_2019_42895_MOESM1_ESM.docx]

**Fixation Characteristics in Highly Myopic Eyes: the Shanghai High Myopia Study**

**Xiangjia Zhu^12345#^, MD, Wenwen He^12345#^, MD, Keke Zhang^12345^, MD, Yinglei Zhang^12345^, MD, Qi Fan^12345^, MD, Yi Lu^12345^*, MD**

1. Department of Ophthalmology, Eye and Ear, Nose, and Throat Hospital, Fudan University, Shanghai, China；
2. Eye Institute, Eye and Ear, Nose, and Throat Hospital of Fudan University, 83 Fenyang Road, Shanghai 200031, People’s Republic of China;
3. NHC Key Laboratory of Myopia (Fudan University), Shanghai 200031, People’s Republic of China;
4. Key Laboratory of Myopia, Chinese Academy of Medical Sciences, People’s Republic of China;
5. Shanghai Key Laboratory of Visual Impairment and Restoration, Shanghai 200031, People’s Republic of China.

*** Corresponding author:**

**Yi Lu**, Eye Institute, Eye and Ear, Nose, and Throat Hospital of Fudan University, 83 Fenyang Road, Shanghai 200031, People’s Republic of China; Tel: +86 21 64377134-407; Fax: +86 21 64318258; e-mail: [luyieent@126.com](mailto:luyieent@126.com)

**# Both authors contributed equally to this work.**


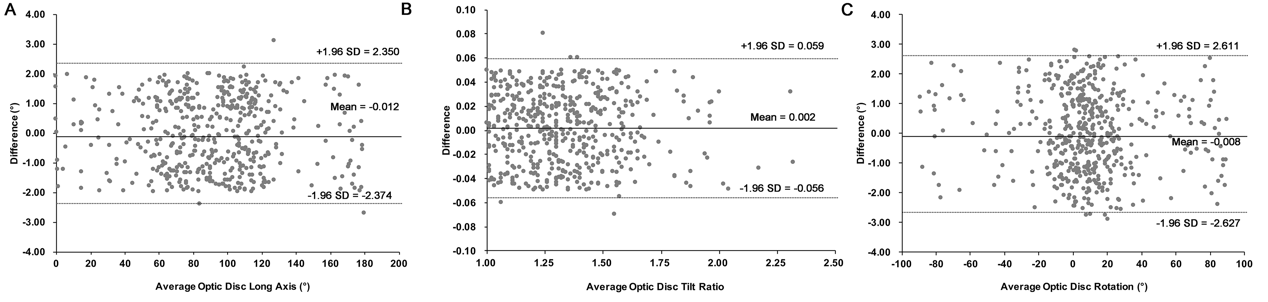
Supplementary Figure 1. Bland–Altman plots showing the agreement in the long axis of optic disc (A), optic disc tilt ratio (B) and the degree of optic disc rotation (C) measured by two researchers. Reference lines correspond to the mean difference and 95% limits of the differences. The agreement in the parameters was good, and there was no systemic bias in the measurements. SD, standard deviation.
